# Exploration of the potential of genomic editing in the treatment of congenital adrenal hyperplasia

**DOI:** 10.3389/fendo.2025.1719376

**Published:** 2025-12-10

**Authors:** Lara E. Graves, Sharntie Christina, Kathryn L. Mullany, Ian E. Alexander, Henrik Falhammar

**Affiliations:** 1 Gene Therapy Research Unit, Children’s Medical Research Institute, Faculty of Medicine and Health, The University of Sydney and Sydney Children’s Hospitals Network, Westmead, NSW, Australia; 2 Discipline of Child and Adolescent Health, Sydney Medical School, Faculty of Medicine and Health, The University of Sydney, Westmead, NSW, Australia; 3 Institute of Endocrinology and Diabetes, The Children’s Hospital at Westmead, Westmead, NSW, Australia; 4 Department of Molecular Medicine and Surgery, Karolinska Institutet, Stockholm, Sweden; 5 Department of Endocrinology, Karolinska University Hospital, Stockholm, Sweden

**Keywords:** 21-hydroxylase deficiency, genomic editing, CRISPR/Cas, AAV, gene editing

## Abstract

Despite life-saving glucocorticoids, therapeutic options for congenital adrenal hyperplasia (CAH) remain sub-optimal. Adrenal crisis continues to be the highest cause of mortality in individuals with CAH and even with recommended treatment regimens complications from the disease and treatments themselves persist. These patients have limited treatment options and advanced therapeutics could be a solution. Development of genetic therapies have exponentially increased in recent years. The advent of CRISPR/Cas technology has brought previously inconceivable treatment options to reality. Genomic editing could repair the defective 21-hydroxylase gene and provide a cure for 21-hydroxylase deficiency, the most common CAH variant, eliminating the current need for constant patient intervention. There are a number of technologies within reach for CAH, however, delivery of the genomic editing reagents to the elusive adrenocortical progenitor cells remains challenging. Here we discuss the complexity of CAH genetics, which has implications for choice of genomic editing strategy, and potential future strategies for the development of a cure of CAH.

## 1 Introduction

Gene therapy technology has reached a critical point where potential curative treatments for monogenic disorders are possible. Congenital adrenal hyperplasia (CAH) is an autosomal recessive monogenic endocrine disorder caused in >95-99% of instances by deficiency of the 21-hydroxylase enzyme (1, 2). CAH can lead to devastating consequences, including adrenal crisis and death, even in the era of glucocorticoid and mineralocorticoid supplementation (3). Current standard management is fraught with inaccuracy and does not completely mimic the physiological profile (4). Undoubtedly, there is a need for a better treatment. Recent progress in genomic therapies have put advanced therapeutics for CAH within reach. While continual turnover of cells in the adrenal cortex obviates standard recombinant adeno-associated virus (rAAV) gene delivery, genomic editing technology could provide a cure for CAH (5–7). Clustered regularly interspaced short palindromic repeats (CRISPR)/CRISPR-associated protein (CRISPR/Cas)-based gene editing technology could be used to repair the defective 21-hydroxylase gene in target progenitor cells of the adrenal cortex (5, 7). Use of non-viral vectors such as lipid nanoparticles (LNP) for delivery of CRISPR can improve the safety of genomic editing and the adrenal gland is an ideal target for LNP (8). While these technological advances have placed precise genetic repair of the 21-hydroxylase locus within reach, reliably targeting the adrenocortical progenitor cells remains a major obstacle, as rAAV vector tropism for these cells is yet to be established. By unravelling adrenal biology, the future could hold robust, durable gene therapy that provides a cure for this devastating disease.

## 2 Congenital adrenal hyperplasia

Congenital adrenal hyperplasia (CAH) refers to a group of seven monogenic adrenal disorders, caused by pathological variants in genes coding for enzymes in the adrenal steroidogenic pathway: *CYP21A2*, *CYP11B1*, *CYP17A1*, *HSD3B2*, *STAR*, *CYP11A1*, and *POR* (9). In 95-99% of cases, CAH is caused by variants in *CYP21A2* resulting in 21-hydroxylase deficiency, and this autosomal recessive disorder affects approximately one in 15,000 livebirths (1, 2). There is a higher incidence in some populations with the incidence in Aboriginal Australians reported as 1 in 6600 livebirths (10), 1 in 3375 in the Roma population of North Macedonia (11) and more than 1 in 300 livebirths among the Yu’pik-speaking Indigenous population in Alaska (12). As deficiency of 21-hydroxylase (P450c21) is the most common form of CAH, for simplicity from here onwards the term “CAH” will refer to this form.

**Figure 1:**
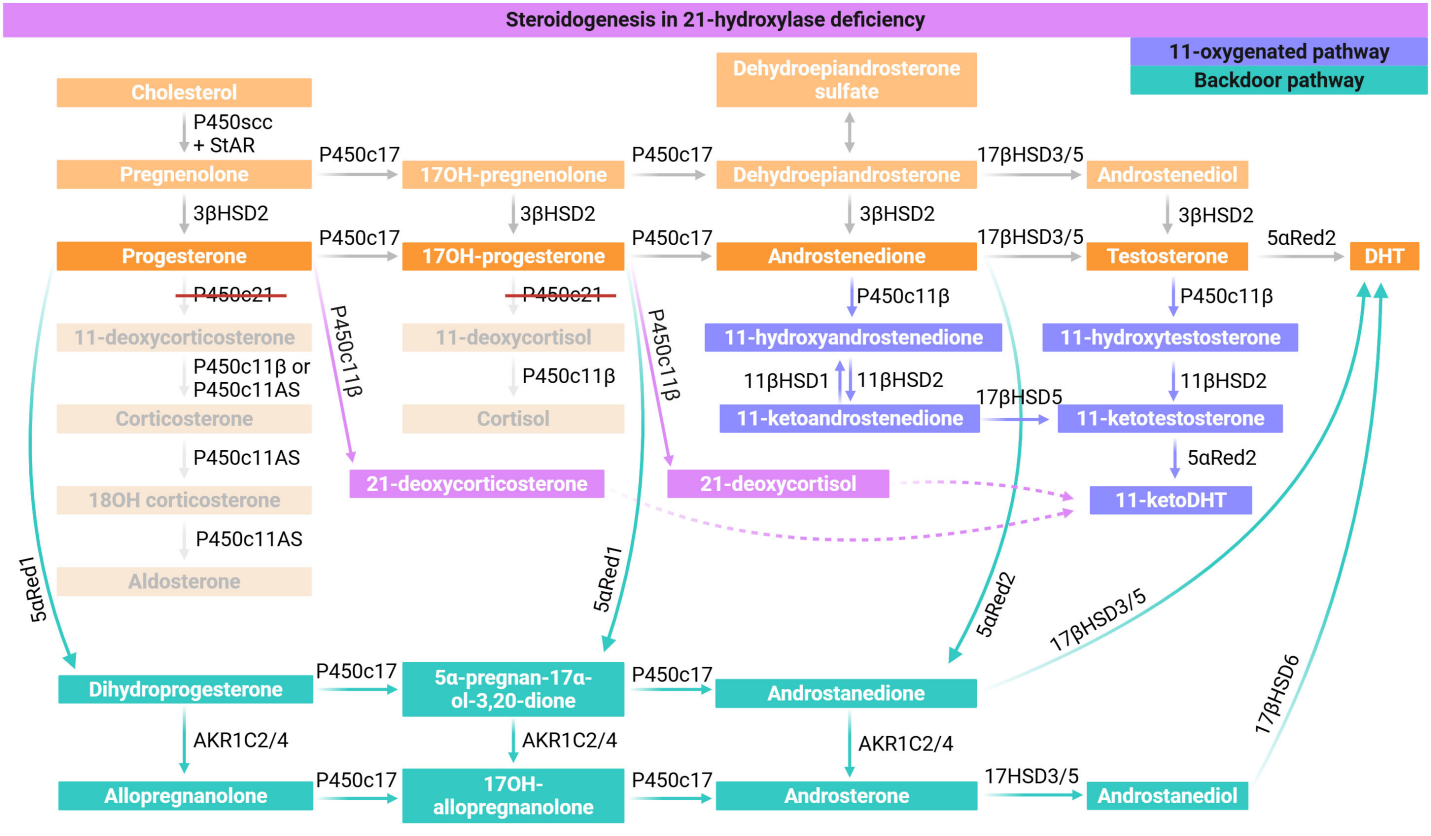
Steroidogenesis in 21-hydroxylase deficiency. As 11-deoxycorticosterone and 11-deoxycortisol cannot be produced in adequate amounts (pale orange), there is a build-up of the precursors (progesterone and 17OH-progesterone) which are then shunted along the canonical androgen pathway via P450c17 (dark orange). Furthermore, there is enhanced activation of the backdoor pathway (green) and 11-oxygenated androgen pathway (blue), further exacerbating the hyperandrogenic state. 21-deoxycorticosterone and 21-deoxycortisol may be produced by P450c11β as specific markers of P450c21 deficiency (purple) and have been proposed to enter the C11-oxygenated androgen pathway.

Pathogenic variants in the *CYP21A2* gene lead to defective 21-hydroxylation and result in a reduction in the synthesis of life-sustaining adrenocortical hormones: cortisol (glucocorticoid) and aldosterone (mineralocorticoid). The upstream precursor steroid hormones accumulate, such as 17-hydroxyprogesterone (17OHP) and these are shunted into the P450c17 pathway, leading to increased concentrations of adrenal androgens via the canonical androgen pathway, the backdoor androgen pathway and through production of 11-oxygenated androgens [**Figure 1**] (9, 13, 14). Lack of cortisol leads to stimulation of the adrenal cortex by adrenocorticotropic hormone (ACTH) and thus to further androgen production. Excess 17OHP may also be converted to 21-deoxycortisol by P450c11β, which has been suggested to be a better marker of P450c21 deficiency than 17OHP, and also provides substrate for the 11-oxygenated androgen pathway (15, 16).

There is a spectrum of disease severity. Phenotypically, CAH can be divided into classical and non-classical (NC) forms, with the classical form further divided into salt-wasting (SW) and simple virilising (SV). Although the phenotypes are defined as such, it is a continuous spectrum based on residual enzyme activity. While aldosterone deficiency is not generally clinically significant in SV and NC CAH, there is some degree of aldosterone deficiency in all phenotypes (17).

As 2% residual enzyme function confers protection from severe SW, it is plausible that delivering the wild-type *CYP21A2* gene to a small number of cells in severe SW CAH could improve the phenotype to SV CAH, or even NC CAH.

### 2.1 Genetic basis of 21-hydroxylase deficiency

There are over 350 known variants in *CYP21A2* listed in the Human Gene Mutation Database (HGMD, http://www.hgmd.cf.ac.uk accessed 4 June 2025) (18) spanning all 10 exons. Therefore, a commercially viable genomic therapy should be designed such that most, if not all, pathological variants can be treated with a single set of reagents to maximise translatability.

**Figure 2:**
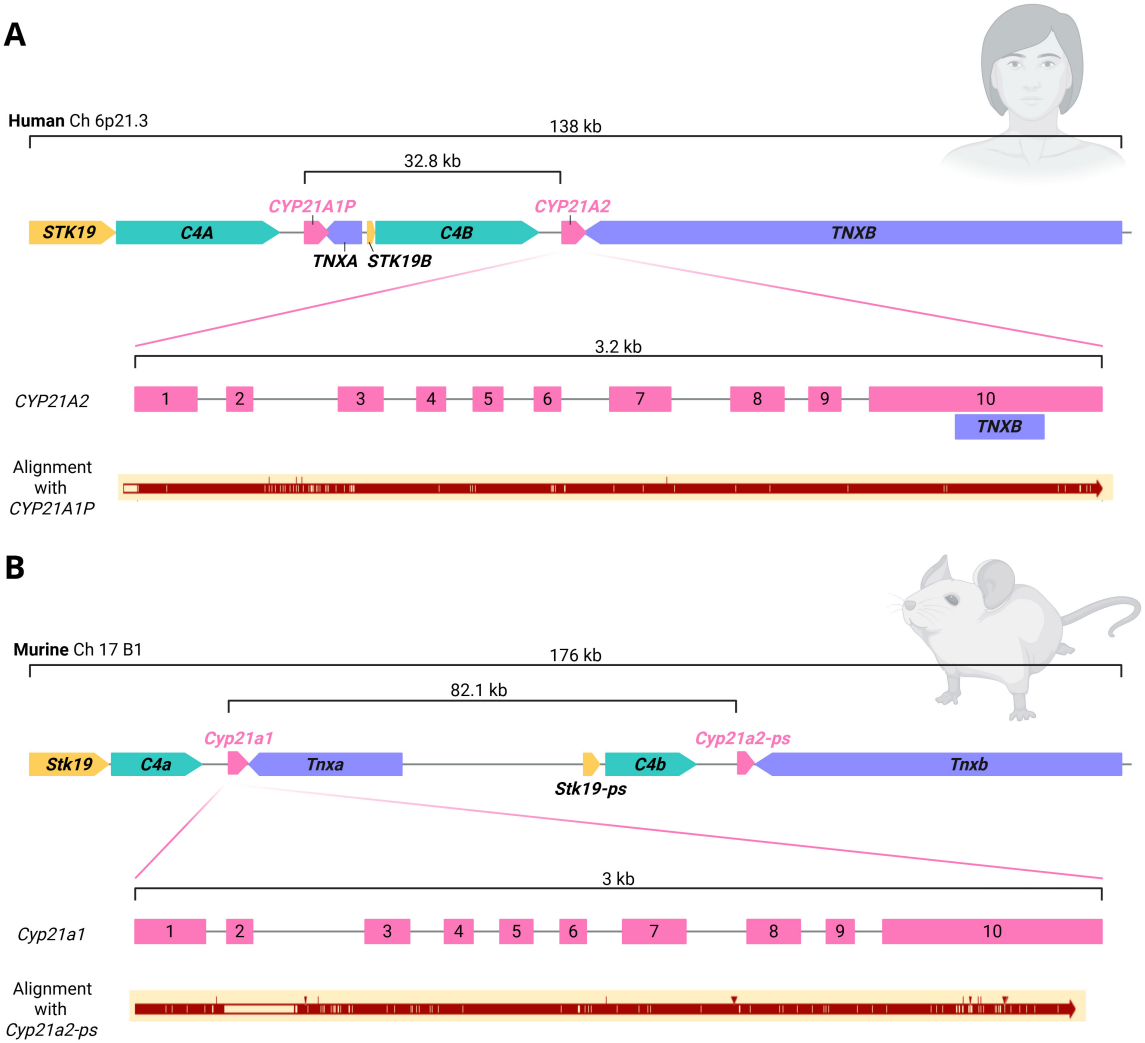
RP-C4-CYP21-TNX (RCCX) module. **(A)** Human RCCX module. A 138 kb region of from the short arm of chromosome 6 is shown (NCBI Accession number NC_000006). There is a tandem arrangement of *C4A*, *CYP21A1P*, *C4B* and *CYP21A2*. The *CYP21A2* gene is 32.8 kb downstream from the *CYP21A1P* pseudogene. The *CYP21A2* locus is 3.2 kb and the cDNA is 2 kb. Alignment with the *CYP21A1P* pseudogene is shown. **(B)** Murine RCCX module. The *Cyp21a1* gene is 82.1 kb upstream from the *Cyp21a2-ps* pseudogene.

The gene for P450c21, *CYP21A2*, spans 3 kb and is located on the short arm of chromosome 6 in the human leukocyte antigen (HLA, also major histocompatibility complex MHC) class III region (19). The segment of the MHC class III region that contains the *C4* and *CYP21A* gene cluster is considered the most complex region of the human genome (20–23). There are at least 14 distinct transcripts in the 140 kb region, and this includes genes that overlap and genes within genes (24, 25). The region is known as the RP-C4-CYP21-TNX (RCCX) module, which is a ~30 kb region that contains 4 genes in close association: serine/threonine kinase 19 (*STK19*, previously *RP*), complement component 4 (*C4*), 21-hydroxylase (*CYP21*), and tenascin-X (*TNX*) [**Figure 2]** (26, 27). This module is usually in duplicate (69% of Europeans) but may also be found as monomodular (17%) or trimodular (14%) copy number variants (9, 28, 29). The duplication borders in the primate and rodent RCCX modules differ, indicating that the duplication event arose separately in these lineages (20). Duplication of the complement 4 gene conferred an evolutionary advantage in immunological function as both genes remain active, allowing variety in the complement C4 protein through differences in gene size, gene number and nucleotide polymorphisms (30). While both the *C4* genes retain biological activity, the remaining duplicated genes have one or more pseudogenes in humans. There is a homologous 21-hydroxylase pseudogene (*CYP21A1P*), 30kb upstream from the active *CYP21A2* and these genes share 98% and 96% homology in the exonic and intronic regions respectively (19, 31). The human pseudogene has multiple variants along the length of the gene and in the promoter region that render it non-functional (19, 31, 32).

**Figure 3:**
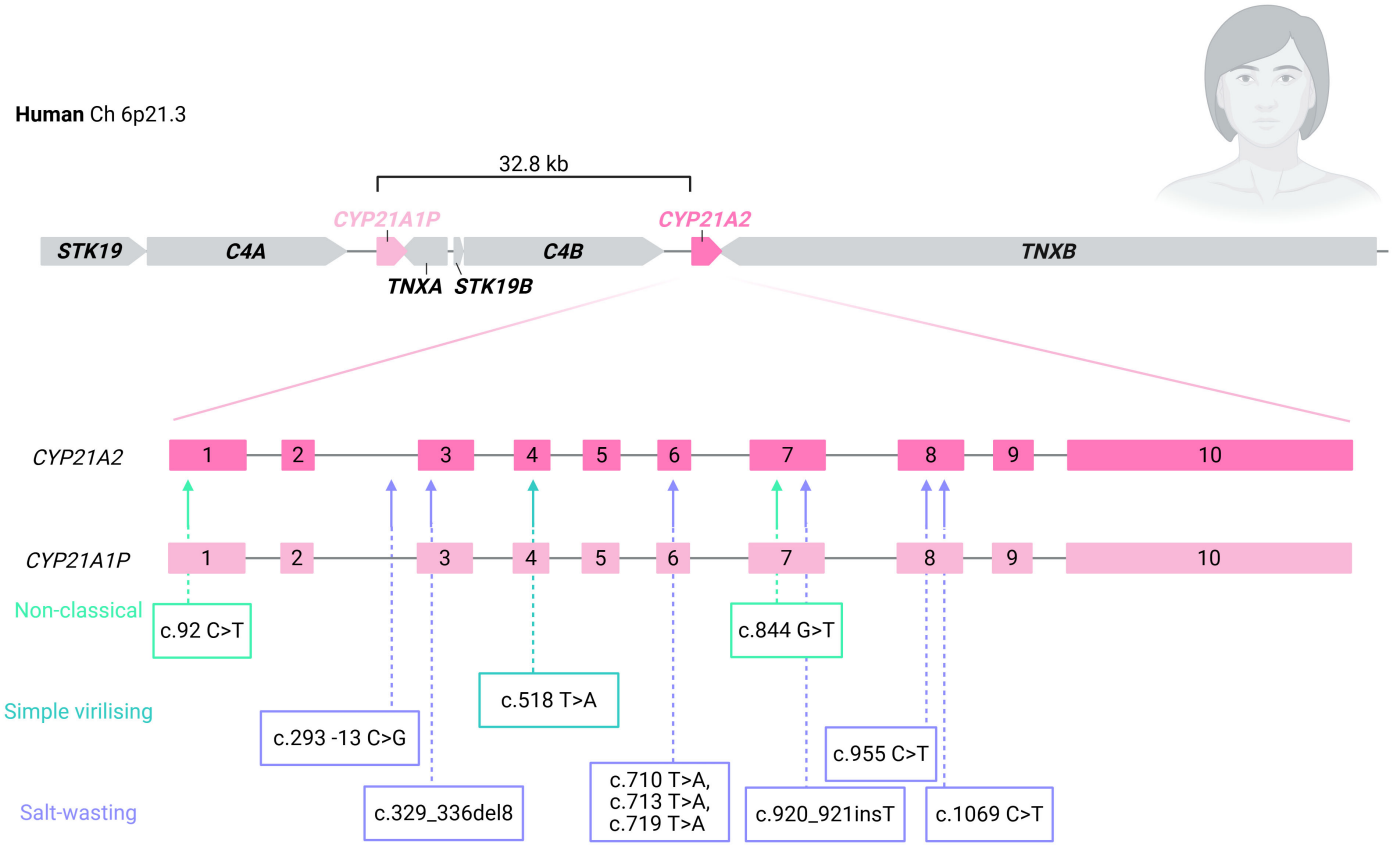
Pseudogene microconversion-derived variants causing 21-hydroxylase deficiency.

**Figure 4:**
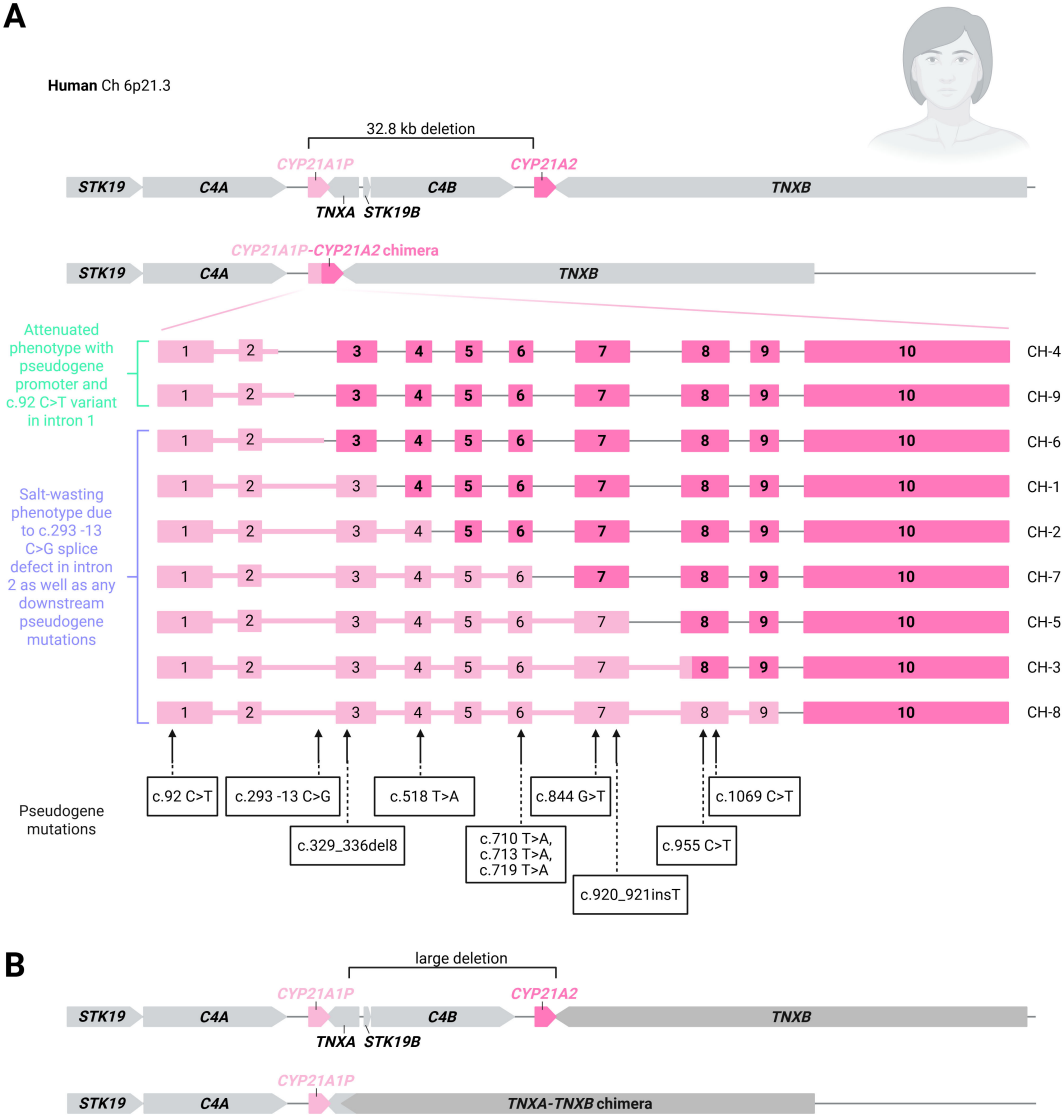
**(A)** Formation of chimeric genes from fusion (CH-1 to CH-9) of the *CYP21A1P* and *CYP21A2* genes. The variants present in *CYP21A1P* are also shown. **(B)** Large deletions may also occur when the TNXA-TNCXB genes form a chimera that deletes the *CYP21A2* gene entirely.

Due to the high homology and tandem-repeat organisation of the genes in the RCCX complex, meiotic recombination events and mitotic gene conversions are common (19, 31, 33–36). Pathogenic variants in *CYP21A2* may occur from misalignment of chromosomes causing unequal crossover, resulting in the formation of large deletions and non-functional chimeric *CYP21A1P-CYP21A2* genes (approximately 20-30%), and gene conversion events resulting in the transmission of pathogenic changes in the pseudogene to the active gene (70-75%) (9, 29, 33, 36–40). Due to this high rate of homologous recombination events that result in genetic alterations 21-hydroxylase deficiency is the most common form of CAH (41–44). Pathogenic variants unrelated to the pseudogene cause a minority (5-10%) of *CYP21A2* variants (29, 45). Allele frequency varies with ethnic background (39, 46). The most common disease-causing variants are due to microconversion events from the pseudogene (36, 47). Pseudogene microconversion-derived variations are listed in **Table 1** and **Figure 3**. Unequal cross-over results in large deletions and chimeric *CYP21A1P-CYP21A2* genes which have been named chronologically by discovery as CH-1 to CH-9 [**Figure 4]** (55). If the breakpoint occurs upstream of the pseudogene intron 2 splice defect (CH-4 and CH-9), individuals tend to have an attenuated (SV or seldom NC) phenotype. However, if the intron 2 splice defect is included in the chimeric gene, then individuals present with classical SW CAH.

**Table 1:** Pseudogene microconversion-derived variants. Nucleotide and codon numbers used are consistent with that listed in the HGMD database (http://www.hgmd.cf.ac.uk accessed 4 June 2025) (18).

Exon/intron	Mutation type	Nucleotide (NM_00500.9)	Codon/protein	Phenotype	Ref
Exon 1	Missense	c.92 C>T	p.(Pro31Leu)	Non-classical	(48)
Intron 2	Splicing defect	c.293–13 A/C>G		Salt-wasting (and simple virilising)	(49)
Exon 3	Small deletion that results in a premature stop at codon 130	c.329_336del8	p.Gly110ValfsX21	Salt-wasting	(41)
Exon 4	Missense	c.518 T>A	p.(Ile173Asn)	Simple virilising (and salt-wasting)	(50)
Exon 6	Complex rearrangement	c.710T>A, c.713T>A, c.719T>A	Ile236Asn, Val237Glu, Met239Lys	Salt-wasting	(49, 51)
Exon 7	Missense	c.844 G>T	p.(Val282Leu)	Non-classical	(51, 52)
Exon 7	Small insertion	c.920_921insT	p.Leu307PhefsX6	Salt-wasting	(41)
Exon 8	Nonsense	c.955 C>T	p.(Gln319*)	Salt-wasting	(53)
Exon 8	Missense	c.1069 C>T	p.(Arg357Trp)	Salt-wasting	(54)

The intron 2 splicing defect is one of the most commonly reported causes of CAH (56). This is particularly the case in populations with Iranian ethnicity (41%) and in the Yu’pik-speaking Indigenous people from Alaska (100%) (46). It was also the most commonly detected variant (34.4%) in a cohort of patients from Australia and New Zealand (39). Large complex deletion/hybrid genes were the second most common reported category of allele in the Australian/New Zealand cohort, and large deletions are commonly seen in individuals with European heritage in other cohorts (46). The variant c.518 T>A; p.(Ile173Asn) in exon 4, was found in 11.3% of screened alleles in the Australian/New Zealand cohort. This prevalence data was consistent with other international studies, with large deletions/hybrids, the intron 2 splice defect and the exon 4 missense variant occurring in the top 3 of most countries with published CAH allele frequency. Japan and China both reported a high prevalence of c.1069 C>T; p.(Arg357Trp), a microconversion from exon 8 of the pseudogene, which was not reported in the Australasian population (39, 57, 58). While a variant-agnostic approach in gene therapy allows the broadest application of a therapy, targeting individual variants can be justified if there is a high prevalence rate. The intron 2 splice site variant is usually associated with a SW phenotype (56), and due to it being one of the most common severe variants, it would be justifiable to develop a gene therapy that specifically targeted this variant. Additionally, while p.(Ile173Asn) in exon 4 is generally associated with SV CAH and may be considered less of a priority, there is phenotypic variability, and it can also be associated with the SW phenotype (37, 39, 59). Therefore, this variant could also be considered for a variant-specific gene therapy. The variants p.(Gln319*), and p.(Pro454Ser) could also be the focus of future work as they are also associated with severe disease with the former reported relatively commonly in Germany, Denmark and Italy and the latter in the UK, Netherlands, Japan and China (39).

Ideally a gene therapy for CAH would treat all gene variants with a single set of reagents. However, this requires insertion of a large DNA sequence at a precise locus which is more challenging than smaller genetic changes, and variants-specific therapies could be considered for the more prevalent small variants.

### 2.2 Current therapies and limitations

While early diagnosis and treatment are imperative in saving the lives of infants born with severe forms of this condition, treatment is far from perfect, and little has changed since steroids were first introduced in the 1950s (60–62). Current standard management is fraught with inaccuracy as there is no perfect biochemical test to monitor therapy (63). Glucocorticoids are required to replace cortisol deficiency and a supraphysiological glucocorticoid dosage is typically required to suppress androgen production. Mineralocorticoids are required in SW forms and may also be used in milder phenotypes (64). Conventional therapies are unable to mimic the physiological requirements and may result in periods of both over- and under-treatment within the same 24-hour period. Too little glucocorticoid can result in adrenal crisis, virilisation (hirsutism, clitoromegaly, disordered menstruation, premature adrenarche), precocious puberty, and short stature; too much glucocorticoid can result in short stature, cardiometabolic effects, decreased bone mineral density and fractures (1, 9, 65–70). There is a constant risk of death from adrenal crisis and in even educated individuals, the risk of death from adrenal crisis is 6% (71). In 187 adults with CAH, the incidence of adrenal crisis was 8.4 per 100 patient-years, and in 38 children with CAH, it was 5.1 per 100 patient-years (72). It may be more difficult to identify adrenal crisis in small children and elderly (73, 74). In children with CAH, the occurrence of adrenal crisis was highest in those 1 to 5 years of age (75). Cortisol is required for the regulation of the adrenal medulla, and relatively reduced catecholamine secretion during adrenal crisis may compound the morbidity and mortality risk in this population (76–79).

It is well recognised that there is increased mortality associated with CAH, even with optimal steroid treatment (3, 69, 70). In a large data linkage study conducted in Sweden, it was shown that individuals with CAH died 6.5 years earlier than controls (3). The two most common causes of death were adrenal crisis (42%) and cardiovascular complications (32%) and included both adults and children in the study. Mental health morbidity is high and also contributes to mortality rates (69, 70). Adrenal crisis was more likely in individuals with SW CAH, indicating that mechanisms to increase endogenous cortisol production and ameliorate the phenotype could be life-saving. While adrenal crisis is a unique risk for patients with adrenal insufficiency, the presence of cardiometabolic risk is common in the general population. However, individuals with CAH have multifactorial increased metabolic risk due to both under- and over-treatment with glucocorticoids, androgen excess, and adrenomedullary failure (66). Increased weight gain, insulin resistance, hypertension, endothelial dysfunction, atherosclerosis and diastolic ventricular dysfunction have all been shown to occur at higher rates in individuals with CAH, contributing to cardiometabolic mortality (66). Furthermore, the SW phenotype had worse outcomes, suggesting that methods to ameliorate the phenotype could improve outcomes (3). None of the current treatment options are able to address the underlying disease process nor reduce the long-term complications of both the disease and its treatment.

As current standard management is far from perfect, and there is no ideal physiological glucocorticoid regimen, alternative and adjunctive treatment options have been explored, including subcutaneous hydrocortisone pumps and modified release twice-daily hydrocortisone (Efmody^®^) (80–84). Neither of these alternatives is ideal as pump management is complex and the modified release hydrocortisone has not been shown to be superior to standard management (85–87). Adjunctive treatment to reduce androgen excess has also been trialled. The enzyme P450c17 converts steroid precursors to DHEA and androstenedione which in turn may be converted to other adrenal androgens. Abiraterone acetate inhibits P450c17 and has been trialled in women with poorly controlled CAH resulting in normalisation of androstenedione concentrations (88). However, abiraterone acetate will inhibit gonadal steroid production and therefore should only be used in pre-pubertal children, women on effective contraception/hormone replacement therapy or men who receive exogenous testosterone replacement (85).

Other strategies to reduce androgen excess include the effect of ACTH excess through either reducing ACTH secretion or blocking the ACTH receptor (84). A selective corticotropin-releasing hormone receptor type 1 agonist (crinecerfont) was approved by the US Food and Drug Administration in December 2024 as an adjunct to glucocorticoid treatment to control hyperandrogenism in adults and children older than 4 years with classical CAH (89). In clinical trials of adults and children, improvement in androstenedione concentrations in those receiving crinecerfont allowed reduction of glucocorticoid dose more than those on glucocorticoid alone (90, 91). There is a Phase II clinical trial utilising a first-in-class oral once daily ACTH receptor (MC2R) antagonist currently known as atumelnant (CRN04894) (NCT05804669), and Phase III clinical trials for paediatric and adult patients are planned commence recruitment in late 2025/early 2026 (NCT07159841 and NCT07144163). By competitively binding and blocking the MC2R, ACTH-mediated steroidogenesis is inhibited, reducing androgen production. Early results demonstrate rapid and sustained suppression of androstenedione and 17OHP concentrations in adults with CAH (NCT05907291) (92, 93). While both of these new treatments may facilitate reduced androgenisation and reduced glucocorticoid dosing, glucocorticoid treatment is still required, and patients are at risk of adrenal crisis if they reduce their dose excessively. These small molecule therapies do not reduce the requirement for constant patient intervention and problems with accuracy of glucocorticoid dosing remain.

Treatment for CAH has changed minimally since it was first applied in the 1950s, despite being far from a perfect remedy. Alternative treatments have been explored but are not superior to standard corticosteroid treatment. Gene therapy is a potentially viable alternative treatment by increasing the P450c21 expression and, thereby, overcoming the deficiency.

## 3 Biology of the adrenal cortex

Since the 1930s it has been known that the adrenal cortex can regenerate itself when only the capsule remains (94, 95), from a population of cells located in the capsule or in the subcapsular region (96). Cells from the peripheral layers proliferate and migrate centripetally until they reach the cortico-medullary junction where they apoptose (97). The cells differentiate into steroidogenic cells in the zona glomerulosa, and during migration, undergo lineage conversion and populate the deeper zones (98). Adrenocortical cellular proliferation is less well characterised in the human than in the mouse, however, cell proliferation is also thought to originate from the periphery of the gland in humans, with the presence of Ki67 staining (a marker for cell proliferation) scattered through the peripheral cortex (99).

There are multiple populations of long-lived stem and progenitor cells in the adult adrenal capsule and cortex. As there is no clear consensus in the definitions, here “stem” refers to the quiescent, long-term retained, multipotent cells that do not express steroidogenic factor 1 (SF1, encoded by *NR5A1*) and “progenitor” cells are those that are actively dividing, with the capacity to differentiate and express SF1+ and sonic hedgehog (SHH) (100). SF1+ embryonic adrenocortical cells give rise to SF1– somatic stem cells that reside as a thin layer in the adrenal capsule (101). Cells expressing GLI1+ are the largest population of capsular cells, which give rise to cortical SF1+ progenitor and SF1+ differentiated steroidogenic cells of the zona glomerulosa (101, 102). Peripheral progenitor cells of the subcapsular zona glomerulosa are characterised by nuclear beta-catenin, SF1+ and SHH+ expression and lack *CYP11B2* expression (98, 102). It is unconfirmed whether these cells are long-term retained (100). SF1+ SHH+ progenitor cells of the zona glomerulosa differentiate into CYP11B2+ steroidogenic cells that produce aldosterone, and then they migrate and undergo lineage conversion to become CYP11B1+ steroidogenic cells of the zona fasciculata that produce corticosterone in the mouse or cortisol in the human (98, 103).

The multiple populations of multipotent adrenal stem and progenitor cells are recruited depending on the physiological or pathophysiological requirement (103, 104). Subcapsular SHH+, SF1+ progenitor cells give rise to most cortical cells (103). The capsular multipotent stem cells are recruited in response to severe stress, but their contribution to the cortex during homeostasis is small (100). The capsular SF1– stem cells also significantly contribute to cell renewal in the adult female mouse adrenal cortex but have an insignificant role in the adult male (105). Male adrenocortical cell renewal relies on SHH+, SF1+ subcapsular progenitor cells (102, 105). Recruitment of the GLI1+, SF1- capsular stem cells is inhibited in the presence of androgens in both male and female mice (105). Capsular cells expressing WT1+ may be recruited in the context of supraphysiological demand (106). Nestin+ cells are scattered in the capsule and throughout the cortex and can differentiate into steroidogenic cells, particularly during times of stress (107, 108). With this constant rate of cell turnover, and renewal, the mouse adrenocortical cells are almost entirely replaced in 200 days (109). This renewal process appears to be faster in female mice than male (3 months vs 9 months) (105). Constant adrenocortical cellular turnover limits the applicability of standard AAV gene delivery for adrenal disorders; therefore, targeted integration of genetic material is required for a robust and durable effect.

## 4 Gene therapy and congenital adrenal hyperplasia

Gene therapy is the use of genes as medicine. This may be through gene replacement, gene silencing or genomic repair. Gene therapies utilise vectors to deliver genetic material either *ex vivo* or *in vivo*. By the first quarter of 2025 there were 33 gene therapies (including genetically modified cell therapies) approved for clinical use in the world, with a further >2000 in development (110). The adrenal gland is likely highly amenable to gene therapy due to properties it shares with the liver such as high relative blood flow and fenestrated endothelium (111, 112). However, constant adrenocortical cellular turnover must be considered when designing a gene therapy approach for CAH.

### 4.1 Gene delivery vectors

Gene therapy requires the delivery of transgenic material into a target cell population. To date, most gene therapies are delivered using viral vectors, exploiting the ability of viruses to deliver their own viral genome into a host cell. However, non-viral strategies such as lipid nano-particle vectors are becoming increasingly popular as they generate weaker immune-responses and can deliver transient RNA or protein cargo (8).

#### 4.1.1 Adenoviral vectors

Adenoviral vectors were popular gene delivery systems early in gene therapy history. Adenovirus is a non-enveloped virus with a double-stranded DNA genome, can package a 26 to 45 kb linear genome and was the gene transfer vector of choice for 15% of gene therapy trials worldwide (113). However, one of the main concerns with adenoviral vectors is immune reaction in the host (114, 115), and their popularity is decreasing due to these adverse effects (116, 117). Furthermore, wild-type adenovirus infection and adenovirus vectors may interfere with adrenocortical function and are therefore a suboptimal choice for adrenocortical disorders (118).

#### 4.1.2 Retroviral vectors

Retroviruses tend to establish a chronic infection, however, there is a risk of complications such as malignancy and immunodeficiency. The lentivirus is part of the retroviral family, containing a single-stranded RNA genome (119). They integrate preferentially into the coding region of genes (119), meaning they could disrupt gene function. Lentivirus does not have specific tissue tropism, and intravenous administration leads to widespread delivery. To target an organ of interest it must be directly administered to that organ. Drug delivery directly to the adrenal gland is technically difficult, and a mouse model for CAH had high mortality when intra-adrenal vector administration was used (120). As the integration of lentivirus into the host cell genome is not controllable and therefore poses additional risks when it comes to off-target insertional mutagenesis, it is most commonly used for *ex vivo* gene editing applications (117, 121). Therefore, this is not a practical choice for the treatment of adrenocortical disease, unless *ex vivo* transduction combined with cell replacement therapy is used, a technology which is not yet developed enough for application to the human context (5, 122).

#### 4.1.3 Adeno-associated virus vectors

There has been a decline in the use of retrovirus and adenovirus vectors in favour of recombinant adeno-associated virus (rAAV) for parenterally delivered gene therapy (116, 123). Recombinant AAV is the leading vector for *in vivo* gene delivery worldwide (113), and in Australia, rAAV-based gene therapy has reached the clinic for the treatment of central nervous system, neuromuscular, retinal and auditory paediatric diseases (124). Without a helper virus AAV can target and enter cells but is considered non-pathogenic (125, 126). Unlike adenoviruses, AAV vectors induce little to no innate immunity, and a far weaker adaptive immune response (127). AAV genomes remain predominantly episomal, although low rates of random genomic integration can occur (128–131).

Recombinant AAV are formed by the removal of most of the viral genome, and replacement with therapeutic transgenic material flanked by AAV2 inverted terminal repeat (ITR) sequences which allow vector genome replication and packaging (123, 132, 133). This vector genome can be packaged into different AAV capsids (a process known as pseudo-serotyping) that show unique cellular tropism, rates of transduction, species specificity, immunological reactivity and packaging efficiency, making them an expansive modular toolkit (124). With appropriate capsid selection, parenteral administration of targeted treatment is possible. The engineering of novel AAV capsid variants by directed evolution and rational design methods has generated libraries of vectors that may perform better for some clinical applications than natural variants (123, 134). The species-specificity of AAV serotypes introduces a limitation when testing rAAV in non-human species, which has implications for pre-clinical experimental design (135).

Both natural infection and exogenous treatment with AAV induces an immune response that cross-reacts with most serotypes. The development of neutralising antibodies following exposure to AAV means that rAAV-based therapy can only be administered once in a lifetime and only to a patient without pre-existing AAV antibodies (136, 137). Strategies to evade this pre-existing immunity such as novel capsid proteins, immunoglobulin (IgG) depletion, immunomodulation, and use of capsid decoys are in development, however such techniques are preliminary and widespread practice is not yet possible (138–140). As such, an AAV based gene therapy treatment must be designed as a “one and done” treatment, providing a robust and durable effect. DNA delivered by AAV exists predominantly in an episomal state, and therefore AAV gene delivery without a reliable method of genomic integration cannot provide a durable effect when targeting cells with high turnover. In post-mitotic organs, such as the central nervous system, muscle or adult liver, AAV episomal DNA may persist for more than 10 years (134, 141). Though during paediatric organ growth, or in organs that maintain lifelong cell turnover such as the adrenal gland, the newly introduced genetic material will be diluted and lost with time (6, 142, 143). Until genetic material can be efficiently and effectively delivered repeatedly, gene addition cannot conceivably play a role in the treatment of CAH or any other monogenic disorder of the adrenal cortex. Genomic editing is therefore poised to supplant gene addition. For a robust and durable gene therapy targeting the adrenal cortex, permanent genomic change must be delivered to the genome of adrenocortical stem or progenitor cells, to ensure that daughter cells maintain the correction.

#### 4.1.4 Non-viral vectors

There is increasing evidence of rAAV-mediated toxicity, including hepatoxicity and thrombotic microangiopathy from parenteral delivery of vector and neurotoxicity from intrathecal delivery of vector (134). Therefore, the gene editing field is moving towards the delivery of Cas9 as mRNA via lipid nanoparticles (LNP), to improve safety profiles (134, 144). Some advantages of LNP over rAAV include large packaging capacity, reduced cost of synthesis, transient expression of delivered RNA material, no risk of RNA cargo integration into the host genome, and reduced immunogenicity (8, 145, 146). However, depending on the editing strategy employed, there may still be a requirement for a viral vector to efficiency deliver DNA to the target cell nucleus, and combination editing therapies using LNP and rAAV are in development. The adrenal cortex is expected to be amenable to rAAV and LNP-mediated delivery of editing machinery due to its high blood flow relative to organ size (111) and fenestrated endothelium (112). Additionally, the high level of lipoprotein lipase receptor expression in the adrenal cortex could facilitate LNP uptake (147, 148). There is mounting evidence that LNP can deliver genetic material to the adrenal gland (144, 149); however, this has not yet been exploited for genetic adrenal disorders (8).

### 4.2 Gene delivery strategies

#### 4.2.1 Gene addition

All but one published attempts to develop gene therapy for CAH have relied on adenovirus- or rAAV-delivered non-integrating gene addition (**Table 2**). If a non-integrating vector is used then the newly introduced genetic material will persist as a transcriptionally active episome, outside of the host genome, and will be lost when the transduced cell divides. This characteristic of AAV has been a major limiting factor in the development of gene therapy for CAH due to constant adrenocortical cellular turnover.

**Table 2:** Gene therapy studies in 21-hydroxylase deficiency: Published pre-clinical gene therapy studies and preliminary results from the human clinical trial.

Study	Model, iIntervention and dose	Outcome					
		Molecular	Enzyme activity	Glucocorticoid	Mineralocorticoid	Progesterone or 17OHP	Other phenotype
1999 (150)	H-2aw18 mouse (151) Adenovirus containing the genomic sequence of human *CYP21A2* delivered by intra-adrenal injection	Highest *CYP21* mRNA expression from days 2–7 and then gradual decline.	21OH activity *in vitro* was undetectable in adrenal glands pre-treatment, and reached wild-type levels in adrenal glands harvested 2–7 days from treatment	Plasma corticosterone increased from undetectable to wild-type levels by 7 and 14 days but declined over 40 days.	Not assessed.	Not assessed.	Improved histological morphology of adrenal gland at 7 days.
2016 (122)	H-2aw18 mouse (151) Genetically modified cell therapy: Tail fibroblasts from 21OH deficient mice transduced *in vitro* with a retrovirus containing murine *Cyp21a1* cDNA and then the cells were implanted subcutaneously.		After *in vitro* transfection of the homozygous fibroblasts, 27.5% of the progesterone added to the culture media was converted to deoxycorticosterone within 24 hours, with negligible conversion in untreated homozygous fibroblasts.	Not assessed.	Not assessed.	Reduction in the serum progesterone-to-deoxycorticosterone ratio in 4/6 mice, 4 weeks after treatment (statistically insignificant).	
2016 (122)	H-2aw18 mouse (151) AAV2 containing murine *Cyp21a1* cDNA delivered by intramuscular injection. Dose: 1×10^11^ vector particles per mouse.	Weak expression in thigh muscle, heart and liver.		Not assessed.	Not assessed.	Reduction in the serum progesterone-to-deoxycorticosterone ratio in 4/4 mice, 4 weeks after treatment (statistically insignificant). Reduction in serum progesterone-to-deoxycorticosterone ratio in a single mouse monitored to 7 months post treatment.	
2017 (152)	H-2aw18 mouse (151) AAVRh10 containing human *CYP21A2* cDNA with a ubiquitous promoter (CAG) delivered by intravenous injection. Dose: 2 ×10^10^ vector genomes per gram of body weight. Mice approx. 15 g at injection therefore 3 ×10^11^ vector genomes per mouse.	18 weeks after transduction vector copies per cell in the adrenal was 0.13 vcn/cell. Reportedly there was even lower vcn in the liver, and no vector expression in the liver. There was low expression in the heart and no expression in kidneys, gonad or brains.		Not assessed.	Small reduction in *Cyp11b2* expression but remained elevated above wild-type. Serum aldosterone concentration not assessed. Renin (*Ren1*) expression reduced 14-fold but did not reach wild-type concentrations.	Reduction in urinary progesterone by 42% at 5 weeks and then remained near normal until 15 weeks (study termination).	Increase in body mass, recovered normal reaction to tail suspension test and improved performance in elevated plus-maze test. No change in adrenal morphology. It was reported that there was decrease in overexpressed *Mc2r*, *Prkar2a*, *Sf1*, *Star*, and *Cyp17a1* genes to near-normal levels, however, on inspection of the figure, although there was a statistically significant reduction in expression of these genes, they did not reach wild-type levels.
2018 (6)	H-2aw18 mouse (151) AAVRh10 containing human *CYP21A2* cDNA with a ubiquitous promoter (CAG) delivered by intravenous injection. Dose: 6.5 ×10^11^ genome copies per mouse.	Transgene expression waned over time: 65% cells in adrenal cortex expressed transgene at 2 weeks, then 41% at 6 weeks, 8% at 16 weeks and 2% at 32 weeks. Substantial liver expression at 32 weeks.		Not assessed.	Not assessed.	Two weeks after treatment serum progesterone reduced from 243 ng/mL to 22.2 ng/mL, which was similar to wild-type levels. This improvement persisted for 6 weeks but then increased.	Serum ACTH concentrations decreased 8 weeks after treatment but increased to pre-treatment levels by 32 weeks.
2024 (153)	H-2aw18 mouse (151) AAV8 containing human *CYP21A2* cDNA with a liver-specific promoter (ApoE/hAAT) delivered by intravenous injection. Dose: 5 ×10^11^ vector genomes per mouse.	Vector in the liver 13 vcn/diploid nucleus in males, 12 vcn/diploid nucleus in females. Vector expression detected in liver, no expression in adrenal gland.		Serum corticosterone increased 4- to 5-fold in treated mice but did not reach wild-type concentrations. 9/10 mice had improvement in whole blood corticosterone from baseline.	Serum aldosterone restored to wild-type concentrations 4 weeks after treatment. Renin expression was reduced to wild-type levels in the treated mice.	Serum progesterone improved in females but did not reach wild-type concentrations and there was no change in the treated males.	Adrenal mass reduced in treated mice but did not reach wild-type sizes. There was no statistically significant difference in serum ACTH, although there was a trend to reduced ACTH after treatment. Expression of *Mc2r* reduced in treated males but not females.
Unpublished, information sourced from press release (154)	Adults with classical CAH BBP-631. AAV5 containing human *CYP21A2* cDNA with a ubiquitous promoter. Dose (155, 156): Level 1 (lowest): 1.5 ×10^13^ vg/kg Level 2 (middle): 3 ×10^13^ vg/kg Level 3 (high): 6 ×10^13^ vg/kg Level 4 (highest): 1.2 ×10^14^ vg/kg.			One treated subject improved their serum cortisol from 105 nmol/L to 235 nmol/L (157). There was increased cortisol production in all patients at “higher” doses.		Majority of patients reduced 17OHP >50%.	At the highest dose, there was an average increase of 11-deoxycortisol by 55-fold.
2025 (7)	H-2aw18 mouse (151) Two-vector system, both packaged in AAV-Rh10: donor vector contained wild-type murine *Cyp21a1* exons 2-10. SaCas9/sgRNA vector contained SaCas9 with a CMV promoter and a guide sequence with a U6 promoter. Both were delivered by intravenous injection. Dose: 1 ×10^12^ vector genomes per mouse (donor vector) and 2.5 ×10^12^ vector genomes per mouse (SaCas9/sgRNA vector)	Editing events were demonstrated with junction of the native exon 1 with the donor exon 2 with 7-8% edited *Cyp21a1* alleles in the adrenal glands 4 weeks after treatment. 5% edited alleles in female mice 15 weeks after treatment Edited transcripts were expressed in adrenal cDNA but not in the liver.		Serum corticosterone increased 6.7-fold in males and 9-fold in females 4 weeks after treatment, and maintained in female mice to 15 weeks (long-term male study not completed). All mice treated with both vectors had improvement in whole blood corticosterone from baseline, which was not seen in the mice that received only one vector.	Serum aldosterone increased above wild-type concentrations 4 and 15 weeks after treatment. Renin expression was reduced to wild-type levels in the short-term mice and suppressed below wild-type in the long-term mice. *Cyp11b2* expression reduced but did not reach wild-type levels.	Serum progesterone reduced 25% in female mice but remained 19-fold above wild-type. There was no change in male serum progesterone.	Adrenal mass reduced in treated mice but did not reach wild-type sizes.

21OH, 21 hydroxylase; CAH, congenital adrenal hyperplasia; vg/kg, vector genomes per kilogram bodyweight; vcn, vector copy number; ACTH, adrenocorticotropic hormone; 17OHP, 17-hydroxyprogesterone.

**Figure 5:**
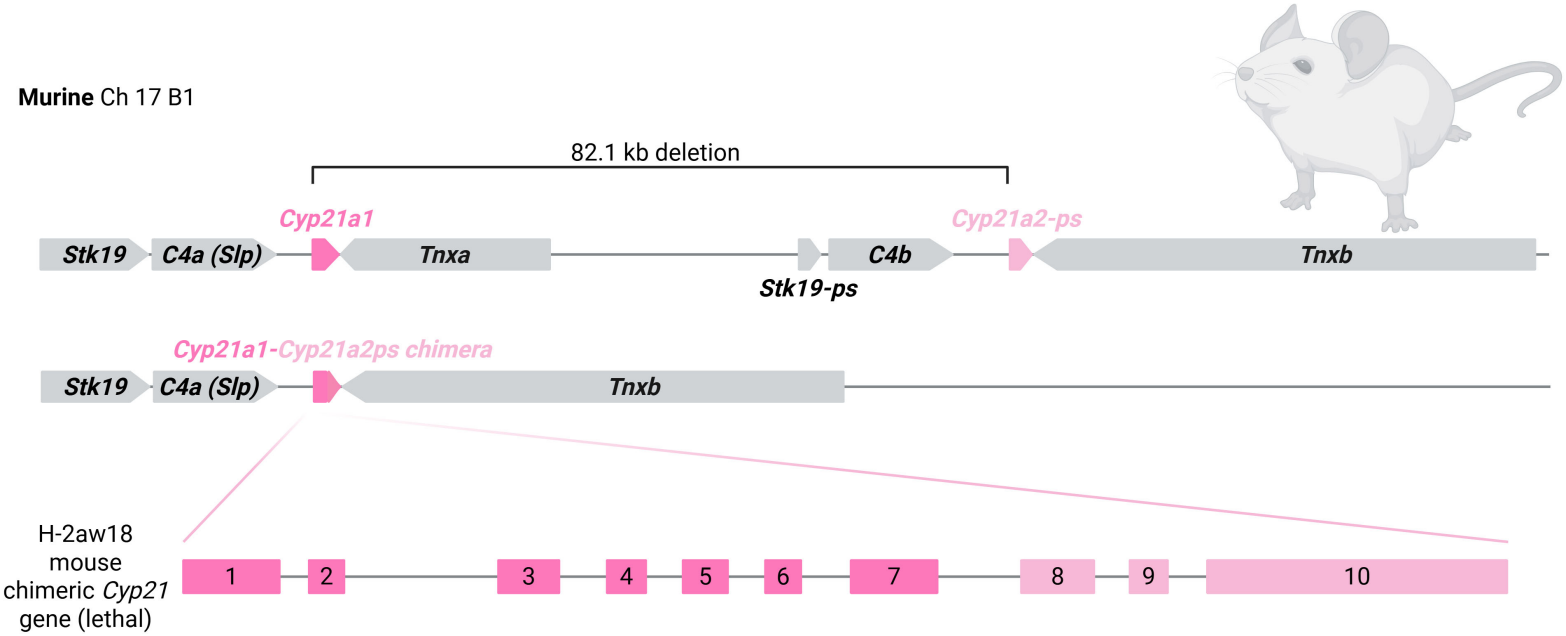
Genetic variant in the H-2^aw18^ mouse used in pre-clinical CAH gene therapy studies. Despite the presence of Exons 1–7 from *Cyp21a1*, this variant is neonatally lethal, consistent with severe salt-wasting CAH.

All pre-clinical studies thus far have used the same mouse model, C57BL/10SnSlc-H-2aw18 (H-2^aw18^) (RRID: *IMSR_NIG:74*), which has 21-hydroxylase deficiency due to a large deletion and formation of a non-functional chimeric *Cyp21a1-Cyp21a2-ps* gene [**Figure 5**] (158, 159). Despite the presence of the first 7 exons of the functional *Cyp21a1* gene, it results in non-functional mRNA and homozygosity for the variant is neonatally lethal, consistent with a severe SW phenotype (151). The mouse may be rescued with exogenous steroids administered to the pregnant dam and neonatal offspring (122, 150, 152, 153, 160, 161). If mice homozygous for the pathogenic allele reach adulthood, they have elevated progesterone, low corticosterone, low aldosterone and enlarged adrenal glands (150, 151, 153).

The first gene therapy for CAH was published in 1999 and used an adenoviral vector to deliver human *CYP21A2* to the H-2aw18 CAH mouse model (150). Mice were injected with vector intra-adrenally to ensure the adrenal gland was transduced. This strategy resulted in the highest *CYP21A2* expression on days 2–7 which conferred phenotypic benefit through improved serum corticosterone and improved the morphological structure of the adrenal glands. However, while the corticosterone peaked on days 7-14, reaching wild-type concentrations, it had declined by 40 days post-injection. Intra-adrenal injections are technically difficult and, when coupled with the fragile nature of the H-2aw18 mouse model, have resulted in high mortality rates, limiting its applicability (120). Furthermore, adenovirus is pro-inflammatory and rAAV is superseding its use in gene delivery (113).

Fifteen years later, in 2016, two alternative strategies were reported: gene-modified cell transplantation and intramuscular gene delivery (122). Fibroblasts from the tails of mice with CAH were obtained and transduced *ex vivo* with a retroviral vector containing murine *Cyp21a1* cDNA. The transduced cells were then subcutaneously auto-transplanted into the mice. While there was a reduction in the serum progesterone to deoxycorticosterone ratio in the transplanted mice, this result was statistically insignificant. Following this, 5 mice were injected with an AAV2 vector containing murine *Cyp21a1* cDNA directly into the thigh muscles, with four mice harvested at 4 weeks, and one at 7 months. All five mice had a reduction in their progesterone to deoxycorticosterone ratio; but this was also statistically insignificant. Furthermore, a slight increase in deoxycorticosterone and a slight decrease in progesterone concentrations could make a large difference in this ratio, while not being a clinically important phenotypic change.

The following year, 2017, the first systemically delivered rAAV gene therapy was published (152). Human *CYP21A2* cDNA was packaged in AAV-Rh10 and delivered intravenously to mice with 21-hydroxylase deficiency. A ubiquitous promoter (cytomegalovirus enhancer element/chicken β-actin promoter/rabbit β-globin intron and splice acceptor, CAG) was used, which would be expected to result in extensive expression throughout the body in all cells transduced by Rh10, although this was not examined by the authors. Despite this expectation, the team detected no expression in the liver, which is unusual as the adult liver is a post-mitotic organ and Rh10 is known to have high transduction efficiency for the murine liver. By 5 weeks there was a statistically significant reduction in urinary progesterone in the treated mice, and although the reduction was maintained to 15 weeks, the difference between treated and untreated mice decreased over time. Both male and female mice were used in all experiments and serum steroids were not assessed. The major steroid excretory pathway in mice is in faeces (162–164) so urinary progesterone may not necessarily reflect serum progesterone. There was also improvement in expression of *Mc2r*, *Sf1*, *Star*, *Cyp17a1* and *Cyp11b2* in the adrenal and *Ren1* in the kidney. The vector copy number detected in the adrenal glands by 18 weeks after treatment was extremely low, at only 0.13 vector copies per cell. Therefore, it is likely that some of the effect still seen at 15 weeks is from vector expressed outside of the adrenal gland. This study provided the first pre-clinical foundation for a human clinical trial.

However, the commencement of this human clinical trial may have been premature (86), as a similar contemporary study demonstrated that the phenotypic effect seen when using rAAV gene addition for CAH was transient (6). In 2018 human *CYP21A2* cDNA packaged in AAV-Rh10 was intravenously delivered to female mice with 21-hydroxylase deficiency. This vector also had a ubiquitous promoter (CAG). After an initial improvement in serum progesterone, an increase from week 6 was observed, with no difference from untreated 21-hydroxylase deficient mice by 10 weeks. Additionally, the rAAV episomes were completely cleared form the cortex by 32 weeks, as expected due to known adrenocortical cellular turnover (105).

An alternative strategy was explored where the human *CYP21A2* cDNA, packaged in AAV8 was delivered systemically for specific hepatic expression (ApoE-hAAT promoter/enhancer element) in mice with 21-hydroxylase deficiency (153). The adult liver is a post-mitotic organ, and this strategy was chosen as a mechanism to avoid the limitations imposed by constant adrenocortical turnover. It successfully increased the serum aldosterone and corticosterone concentrations but was unable to achieve a reduction in progesterone. High progesterone concentrations may be required to drive the remote 21-hydroxylation. Furthermore, while corticosterone production improved, serum concentrations did not reach wild-type levels for each sex. If applied to the human context, glucocorticoid cover would still be required at times of physiological stress, and methods to reduce hyperandrogenism would be required. While this strategy has the potential to alleviate the severity of the SW phenotype in adults with CAH, it does not provide a complete cure.

Understanding of the biology of AAV and the adrenal gland is imperative when designing a gene therapy for CAH. Clearly, some earlier methods did not address the limitations imposed by adrenocortical cellular turnover nor was this appreciated prior to the commencement of the clinical trial. The gene therapy developed was AAV5 containing human *CYP21A2* with a ubiquitous promoter, delivered intravenously to adults with CAH. At the time of trial closure, adults with CAH had received the BBP-631 vector at 4 doses (**Table 2**). While there was some improvement in glucocorticoid production at the higher doses, the results were not considered adequate to justify continuation of the trial and further development of this product was abandoned (154, 165). The adrenocortical cell turnover time in humans is unknown. Use of AAV5 and a ubiquitous promoter would have resulted in expression of *CYP21A2* throughout the body, and the effect seen on glucocorticoid concentrations could have been a result of ectopic 21-hydroxylation, if the adrenocortical cell turnover time in humans had been exceeded. This trial serves as a reminder that AAV-based gene therapy is not an off-the-shelf product, and the biology of the vector and the living system it is being introduced into must be carefully considered. Future CAH gene therapy must focus on strategies to create a durable effect, if AAV is to be used, or methods whereby repeated dosing may be facilitated.

#### 4.2.2 Gene transfer with transposases

One alternative technique explored to improve the durability of AAV gene transfer has been through the use transposons (166). Transposons are a naturally occurring ancient group of genes that can manipulate segments of chromosomal DNA in a manner distinct from other editing endonucleases and are implicated as having an important role in vertebrate evolution (167, 168). ‘Transposon’ refers to the genetic material that is being inserted, ‘transposases’ refer to the nuclease enzymes or moiety that moves the DNA and ‘transposable elements’ refer to any moiety that is involved in this process (169). Recently, bioengineering of eukaryotic DNA transposon systems have also been exploited for specific gene therapy uses, with the most commonly used DNA transposases being Sleeping Beauty and *piggyBac* (170–172). These platforms have been used in AAV gene addition approaches to improve the persistence of transgenes by utilising the natural insertional ability of these transposase enzymes in tissues with high cellular turnover (173, 174). Transposases like *piggyBac* achieve transposition through a series of transesterification and hydrolysis steps that generate an excised intermediate with transposon ends protected by DNA hairpins (175). This process occurs at sites rich in the specific tetranucleotide repeat, TTAA, and leaves no excision footprint. Sleeping Beauty transposase is a bioengineered synthetic transposase and whilst follows a similar “cut and paste” mechanism to *piggyBac*, leaves a TA dinucleotide duplication footprint at the site of integration and excision (176).

While a DNA integration approach for CAH using conventional transposable elements could allow persistence of expression of the newly introduced *CYP21A2*, the site of integration of the transposable element is random and would not allow for physiological control of gene expression through transcriptional regulation under the native *CYP21A2* promoter. Instead, control of 21-hydroxylation would be indirect through physiological control of precursor synthesis, an imperfect method of enzymatic control. Furthermore, as integration with *piggyBac* and Sleeping Beauty transposases is random, there is a theoretical risk of insertional mutagenesis and genotoxicity associated with their use. Additionally, a further limitation of this strategy for gene therapy use is the packaging limitation of rAAV. Due to the size of the transposase coding sequences, dual vectors are required in this strategy, one rAAV to deliver the transposase and another to deliver the therapeutic transgene. Co-transduction of the same cell with two vectors undoubtably introduces an additional element of complexity to any therapy moving towards the clinic. There are no clinical trials currently using transposases in gene delivery *in vivo*. Development of lymphoma after *ex vivo piggyBac* modified CAR-T cell treatment has been reported, raising important safety concerns about this technology (177, 178), although the authors postulated that unique CAR-T production methodology caused the multiple integrated transposons and chromosomal rearrangements seen in the transformed malignant cells, rather than the use of *piggyBac* transposase itself. Undoubtedly, the bioengineering of these ancient genes has added an additional molecular technique to the growing armamentarium of gene therapy platforms but requires further pre-clinical study with regards to safety before clinical application.

#### 4.2.3 Gene editing

A limitation of rAAV gene addition approaches is that the transcriptionally active episome is lost during cellular replication (142). This is particularly important in the paediatric population where rapid growth through cell division is taking place (143). However, there are organs where cellular populations continue to turnover throughout adulthood, such as in the adrenal cortex. Furthermore, gene expression by gene addition is not controlled by the genes’ native promotor, thus true physiological control of gene expression is not possible.

Gene editing is the process by which a targeted change is introduced into the host genome at a specific locus which is heritable to daughter cell populations. A number of early technologies based on programmable nucleases including meganucleases, zinc-finger nucleases, and transcription activator-like effector nucleases (179, 180) have now been superseded by CRISPR/Cas (181). CRISPR/Cas endonucleases, discovered to exist as a bacterial defence system against invading viral DNA, are capable of being programmed to target a DNA locus by simple Watson-Crick base pairing with guide RNA (gRNA) sequences, a process which is far simpler than its predecessors in gene editing technologies (182). The CRISPR/Cas9 endonucleases cleave DNA by pairing of the gRNA to the matching genomic target region and recognition of an upstream protospacer-adjacent motif (PAM) (182). Double stranded DNA cleavage occurs simultaneously, creating blunt ends for repair (183). By inducing targeted double-strand breaks (DSBs) in the genome, pre-existing cellular DNA repair pathways are activated which repair the break and can integrate site-specific modifications (184).

The main DNA repair mechanisms utilised in gene editing are non-homologous end joining (NHEJ) and homology-directed repair (HDR) (185–189). The most active double-stranded DNA break repair pathway in mammals is NHEJ, however, it is error prone and may introduce insertions/deletions (indels) which could disrupt gene function (190). While HDR is limited to actively dividing cells and therefore occurs less frequently, it allows for precise repair (191). Gene editing using HDR may be limited by the presence of single nucleotide polymorphisms (SNPs) and other genetic variation around the target site due to the reliance on homology arms, and the inserted gene sequence is generally limited to 1–2 kb in size (192). These limitations render HDR an inappropriate choice for a universal editing approach for the *CYP21A2* locus in the adrenal gland.

Gene editing for CAH has great potential, as it could ameliorate the phenotype or possibly cure 21-hydroxylase deficiency by repairing the defective gene and allowing physiological control of expression. The highest cause of mortality in individuals with CAH is adrenal crisis (3), and even partial restoration of corticosteroid production could reduce this risk. However, delivering the gene editing reagents to the progenitor cells is required for a durable effect, and establishment of vector serotypes that can efficiently transduce this elusive population is required. Furthermore, optimization of safety, specificity and editing efficiency is required.

##### 4.2.3.1 Homology-independent targeted integration

**Figure 6:**
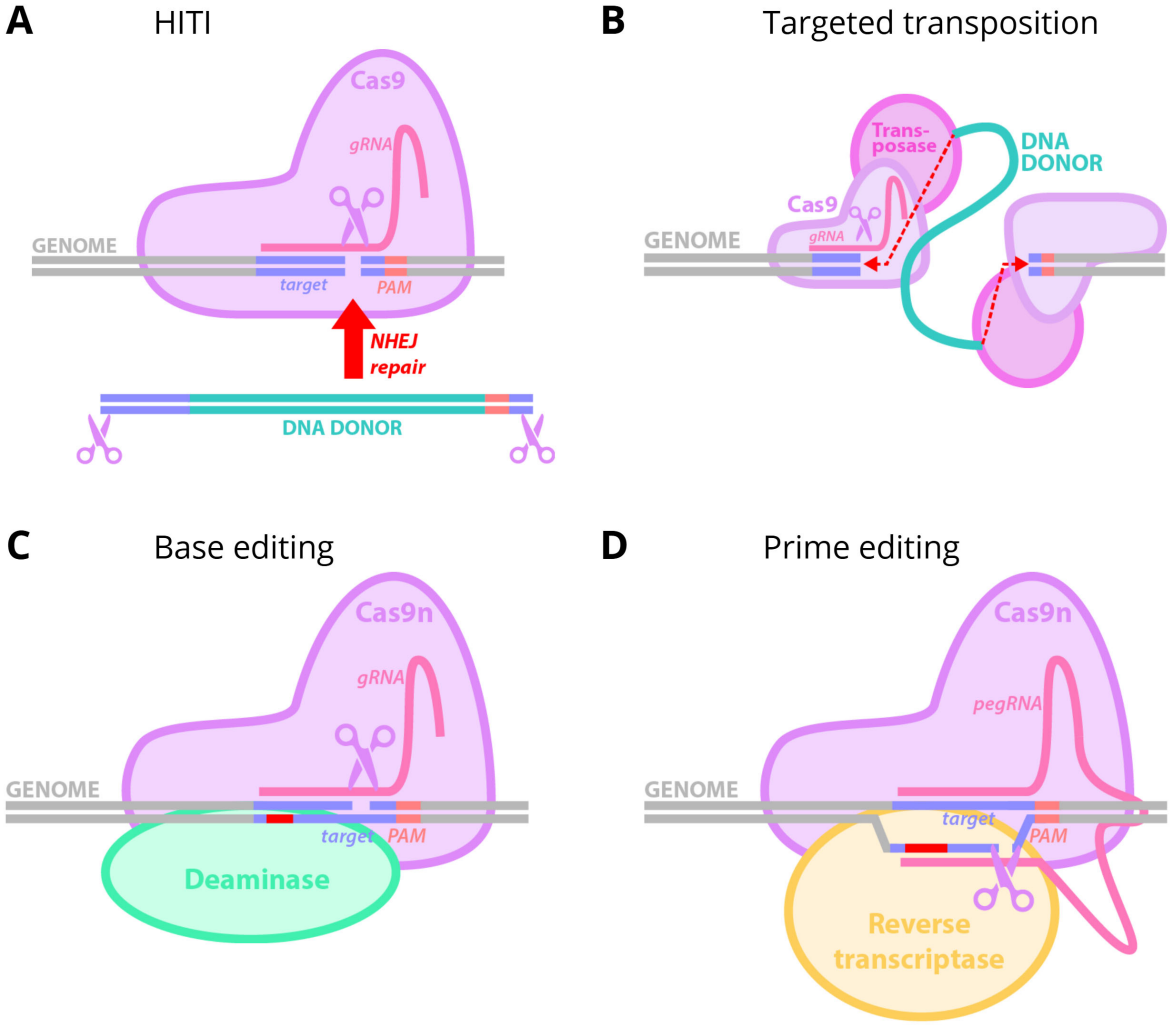
CRISPR-based editing techniques. **(A)** Homology independent targeted integration (HITI). **(B)** Targeted transposition. Transposon-mediated ligation shown in red. **(C)** Base editing. Edited nucleotide shown in red. **(D)** Prime editing. Edited nucleotides shown in red. Abbreviations: Cas9, CRISPR-associated protein 9; Cas9n, Cas9 nickase; gRNA, guide RNA; PAM, protospacer adjacent motif; NHEJ, non-homologous end-joining; pegRNA, prime editing guide RNA.

Homology-independent targeted integration (HITI) is designed to rely on NHEJ to overcome the limitations of HDR repair (193). The use of targeted CRISPR/Cas9 machinery ensures the host genome is precisely cleaved. Similarly, CRISPR/Cas9 creates DSB in provided donor DNA sequences. With the help of NHEJ, the donor sequence is then integrated into the host DNA, between the guide sequence and the PAM site [**Figure 6A**]. For adrenocortical disorders, the genomic editing machinery must be delivered to adrenocortical stem/progenitor cells, so that the gene change is inherited by daughter cells and which will allow for a durable effect. In CAH, the wild-type *CYP21A2* exons 2–10 could be integrated into intron 1, resulting in a fusion of the host exon 1 with the donor exons 2-10, which would allow correction for all downstream variants (7). Using a strategy such as this, the majority of deleterious variants in *CYP21A2* could be corrected with this single set of reagents. For upstream variants, an internal ribosome entry site (IRES) could be used to allow the entire *CYP21A2* +/- promoter sequence to be integrated. Ideally, if the donor sequence is integrated in the reverse orientation, the guide sequence and PAM sites re-align, and CRISPR/Cas9 will cleave the donor sequence out again. If the donor sequence is integrated in the correct position, the guide sequence and PAM sites do not align, and no further cuts occur (183). In practice, indels introduced at the integration site by NHEJ repair may eliminate the target recognition site, leaving reverse orientated donors *in situ*. Furthermore, a number of unexpected integration events have been increasingly recognized as taking place using a HITI methodology, including integration of subgenomic fragments from the Cas9 vector and sections of ITRs. These non-functional inserts will compete with the correctly oriented insert, reducing efficiency. While HITI could be applied to the *CYP21A2* locus for durable treatment of CAH (7), unintended genomic events with this technology are frequent (194–200) and strategies need to be developed to improve safety and precision before application to the human context.

While in the following study the adrenal was not the target organ, it is the first demonstration that adrenal cell genomes are amenable to editing (201). Adrenoleukodystrophy is a genetic condition due to pathogenic variants in the *ABCD1* gene which results in multi-organ pathology. A pre-clinical genomic editing study used rAAV9 to deliver editing machinery in a mouse model for adrenoleukodystrophy at a dose of 1×10^12^ vg/mouse (total vector which included both the CRISPR/Cas9 vector and the donor vector, but the ratio of each vector was not described). Capsid serotype AAV9 was chosen for the ability to transduce both the central nervous system and the liver. AAV9-CRISPR/Cas9 delivered systemically resulted in 7% indels in the liver and 1% indels in the adrenal gland, but indels were not detected in other organs. The HITI cassette was detectably integrated in genomes extracted from the adrenal gland. Using two rAAV9 vectors to deliver CRISPR/Cas9 and a HITI repair cassette systemically, the *ABCD1* gene was edited in both the liver and adrenal. While this study is the first to demonstrate *in vivo* editing of murine adrenal, adrenal editing was not its main goal and the study did not account for the adrenocortical turnover.

A recently published article from our laboratory demonstrated editing of the 21-hydroxylase locus in the adrenal gland *in vivo* as a therapeutic approach to CAH (7). In this study a HITI strategy was used to target a cut site in intron 1 of *Cyp21a1* in the H-2aw18 mouse model and integrate a donor cassette containing a splice acceptor and codon-optimised wild-type exons 2-10, effectively repairing the native locus and restoring physiological control of gene expression. AAV-Rh10 was used to deliver the editing machinery systemically, and this serotype was chosen due to previous efficient gene delivery to the murine adrenal gland (6, 152). This strategy used a higher vector dose than the adrenoleukodystrophy study, and resulted in 5-12% edited alleles in the adrenal gland and 11-24% edited alleles in the liver (7). There was resultant improvement in the phenotype (**Table 2**). All mice that received both vectors had improvement in their phenotype, which was not seen in those that received a single vector, indicating the phenotypic effect was from gene editing, not the presence of the donor vector alone. This study also indirectly demonstrated potential editing of the adrenocortical progenitor cells, as evidence of the edited allele and the phenotypic effects persisted to 15 weeks in female mice, which is 3 weeks after the female murine adrenal cortex is expected to completely renew (105). This demonstrates that AAV-Rh10 may be able to transduce stem or progenitor cells in the murine adrenal cortex, however this does not necessarily translate to human adrenocortical cells as AAV capsid tropism is species-specific. While this study demonstrated that the *Cyp21a1* locus is amenable to editing and can result in a durable benefit, there are significant challenges that need to be surmounted prior to human translation, which include the development of a humanised adrenal model system that can allow development of human-specific reagents.

##### 4.2.3.2 Targeted transposition

Early efforts to develop an ability to target transposition were attempted through fusion of sequence-specific DNA binding proteins (eg zing-finger proteins, TAL effectors and dCas9 fusions) with transposases, however they had limited efficiency and precision (202–205). CRISPR-associated transposons (CASTs) were discovered in bacterial genomes which are composed of a transposase complex and a CRISPR effector, guided by RNA (206–208). Emerging technologies using CASTs are now being explored to allow targeted insertion of large DNA cassettes using minimal CRISPR systems for RNA-guided DNA transposition, without DNA cleavage (206–208). In the absence of DNA cleavage, safety is improved as there is less risk of unexpected DNA rearrangement events. The exact mechanism of action is still under investigation (209, 210).

A strategy that combines both the target selectivity of CRISPR with transposable elements has been developed known as Transposase-CRISPR mediated targeted integration (TransCRISTI) (211). The system utilizes a fusion of Cas9 nuclease with a *piggyBac* transposase and has high homology-independent editing efficiency *in vitro*. DNA excision is performed by the *piggyBac* transposase and the site-specific integration by Cas9 [**Figure 6B**]. Re-integration of the transposon into another region of the genome is prevented by the introduction of variants into the *piggyBac* sequence, and the transposon element is then tethered for integration by Cas9. However, increased precision is associated with decreased efficiency of gene delivery (212), and to overcome the hyperandrogenism seen in CAH, it is likely that a high percentage of cells will require correction.

##### 4.2.3.3 Base editors

Base editors allow for precise single nucleotide genomic change without the requirement of repair of double-stranded breaks which avoids the risk of inducing indels associated with DNA repair (213). Adenosine base editors change A/T to G/C and cytosine base editors change C/G to T/A (213, 214). Catalytically impaired CRISPR/Cas9 is fused with a DNA deaminase enzyme, and this complex is able to be programmed with a guide RNA [**Figure 6C**]. Base editors are highly efficient and allow programmable repair or point variants without double-stranded DNA cleavage, without the requirement for donor templates and without the reliance on unpredictable DNA repair (215, 216).

Of the 359 known causative variants in CAH, 58% are single nucleotide variants (HGMD, http://www.hgmd.cf.ac.uk accessed 4 June 2025) (18). While single nucleotide variants may be amenable to base editing, a unique set of reagents is required for each variant. If base editing were to be used, this would require over 200 unique sets of reagents, which is not commercially viable. However, focused attention could be given to the most common and severe single nucleotide variants, such as those that are derived from the pseudogene during microconversion events. Examples include the pathogenic variants in exon 8 that cause SW CAH comprising the nonsense c.955 C>T and missense c.1069 C>T where an adenosine base editor may be able to edit the T and replace with C, pending the requirement for a local recognition (PAM) sequence. Similarly, a cytosine base editor may be amenable to treating the intron 2 splice defect c.293–13 A/C>G, where G could be replaced with A.

The use of base editors would eliminate the requirement for unpredictable DNA repair mechanisms, and donor templates. This may improve editing efficiency for specific variant types.

##### 4.2.3.4 Prime editors

Prime editing is a CRISPR-based technology that allows for “search-and-replace” modification of the genome (217). The prime editor protein is a fusion of Cas9 nickase and reverse transcriptase and allows the introduction of targeted DNA changes without double-stranded DNA cleavage [**Figure 6D**]. The system is programmable through the use of a prime editing guide RNA (pegRNA). This strategy limits the possibility of unintended events due to the absence of a double stranded DNA break (217). Unlike base editing, which is limited to four possible base substitutions, prime editing enables all 12 nucleotide conversions for point variants and small nucleotide sequence insertions. However, current prime editing technology is limited to small DNA changes due to decreasing insertion efficiency with cargo size, thus insertion of an entire gene coding sequence is not plausible. Furthermore, efficiency varies between organ and cell systems, and optimization is required. Similar to base editing, prime editing could be used to target specific, common disease-causing variants, but would not provide a variant agnostic approach to CAH.

##### 4.2.3.5 Other editing strategies using Cas9 nickase

As the limitations of HITI are increasingly recognized, strategies to introduce large genomic modifications are required. The use of Cas9 nickase, which can allow targeted genomic change without double stranded DNA break, is being increasingly exploited for this purpose. Strategies such as programmable addition via site-specific targeting elements (PASTE) (218) and phage-assisted continuous evolution enhances prime-editing-assisted site-specific integrase gene editing (PASSIGE) (219) show promise.

### 4.3 Safety of editing the genome

The first CRISPR-based gene therapy was approved for use in the clinic for treatment of the haemoglobinopathies sickle cell disease and transfusion-dependent ß-thalassaemia (220). This strategy is a non-viral cell therapy and works by disrupting expression of *BCL11A* which is required for the suppression of foetal haemoglobin expression. By increasing foetal haemoglobin expression, which is biologically functional, this reduces anaemia and painful sickling attacks. While this approval, which comes only 11 years after the discovery of the CRISPR/Cas system (182), paves the way for other CRISPR based therapies, knocking out a gene’s function is much simpler than repairing a gene.

Cleavage of DNA by CRISPR/Cas9 is targeted to the genomic loci by homology of the gRNA molecule. Off-target loci within the genome, especially those that share homology with the gRNA sequence, can become sites of unwanted CRISPR/Cas9 cleavage. Off-target cleavage can lead to disruption of important genes, insertional mutagenesis, or other unwanted genomic aberrations.

In addition to off-target effects, on-target unexpected events at the DNA cleavage site such as large deletions, large integrations of sub-genomic fragments from either the Cas9 or donor vector and chromosome rearrangements are increasingly recognised as risks associated with genomic editing (7, 194–198). When DNA is delivered by AAV it may be retained in a non-dividing cell for years (221, 222) and Cas9 delivered as DNA will require promoter sequences to facilitate expression. Long-term expression of Cas9 may cause prolonged editing events. Furthermore, there is a risk that these strong promoter-enhancer sequences may be unexpectedly integrated, with the potential for oncogenesis through the dysregulation of nearby genes.

Other safety concerns that must be addressed include consideration of immunogenicity of production of a protein of which the subject has not previously had endogenous production, and inadvertent editing of germ cells. These effects can have serious consequences and must be thoroughly considered and minimised prior to the use of these technologies in humans.

## 5 Conclusion

Gene editing has the potential to provide a robust and durable cure for CAH if the elusive adrenocortical progenitors were targeted. Alternatively, there is a need for a delivery system that is amenable to repeat dosing to overcome the loss of edited genomes in differentiated adrenocortical cells, associated with cell turnover. Challenges remain in repairing large DNA sequences with high efficiency and minimal off target effects. Bespoke variant specific treatments may be within reach, however, may not yet be commercially viable.
